# Thymic Squamous Cell Carcinoma: A Population-Based Surveillance, Epidemiology, and End Result Analysis

**DOI:** 10.3389/fonc.2020.592023

**Published:** 2020-12-22

**Authors:** Xudong Yang, Kejia Zhao, Chuan Li, Yanbo Yang, Chenglin Guo, Yi Pu, Lunxu Liu

**Affiliations:** ^1^Department of Thoracic Surgery, West China Hospital, Sichuan University, Chengdu, China; ^2^Western China Collaborative Innovation Center for Early Diagnosis and Multidisciplinary Therapy of Lung Cancer, Sichuan University, Chengdu, China

**Keywords:** thymic carcinomas, thymic squamous cell carcinoma, SEER database, complete resection, prognosis

## Abstract

**Objectives:**

Thymic squamous cell carcinoma (TSCC) is a rare neoplasm that has been sparsely cited in the literature. The aim of this study was to determine disease characteristics and prognostic factors of patients in a Surveillance, Epidemiology, and End Results (SEER) analysis.

**Methods:**

Cases from 1990–2016 were retrieved from the SEER database and demographics, treatments, and survival outcomes were analyzed.

**Results:**

The TSCC accounted for 72.4% of the thymic carcinomas and 7.2% of thymic tumors. The 276 patients (165 men) selected for analysis had a median age of 65 (24–85) years, and 201 patients were diagnosed with Masaoka-Koga stage III/IV. The median survival of TSCC was 59 months with a 49.0% 5-year OS rate, a better prognosis than lymphoepithelioma-like carcinoma (32.1%) and undifferentiated carcinoma (33.3%). Multivariate analysis revealed the Masaoka-Koga stage (p = 0.003) and surgical types (complete resection, incomplete resection, and none; p < 0.001) were determinants of survival. Complete resection had the best prognosis with a 72.7% 5-year OS rate. Chemotherapy was an independent protective factor (HR = 0.555, 95% CI 0.347–0.886; p = 0.014) though poor survival was showed in univariate analysis. And the survival benefit of chemotherapy was validated in PSM analysis (3-year OS rate was 77.7% with chemotherapy *vs.* 52.8% without chemotherapy; p = 0.014).

**Conclusions:**

TSCC was frequently diagnosed in older patients with advanced Masaoka-Koga stage and had more favorable survival than other subtypes of thymic carcinomas. Complete resection is the preferred treatment. Masaoka-Koga stage and chemotherapy had a strong association with prognosis.

## Introduction

Thymic neoplasms are rare tumors that account for less than 1% of all adult cancers (1). Thymic epithelial neoplasms are major thymic neoplasms subtypes that comprise thymomas and thymic carcinomas. The outline of thymomas has been well clarified compared with thymic carcinomas as its advantage on larger cases. Different from the tumors of other sites using the TNM stage system, the most widely accepted clinical staging system in thymoma is the Masaoka stage. And was developed by Masaoka *et al*. in 1981 and modified subsequently by Koga et al. in 1994. The Masaoka stage was also applied in thymic carcinomas in many series (2, 3). Thymic carcinomas represent a heterogeneous group of tumors that exhibit more aggressive malignant behaviors compared to thymomas (4).

According to the World Health Organization (WHO) criteria of histologic classification, Thymic carcinomas consist of over 10 subtypes (5). Some subtypes such as thymic squamous cell carcinoma (TSCC, major subtype), Basaloid carcinoma, mucoepidermoid carcinoma, lymphoepithelioma-like carcinoma, clear cell carcinoma, sarcomatoid carcinoma, adenocarcinoma, and undifferentiated carcinoma were recorded in different proportions among the series of thymic carcinomas studies. Each histologic subtype has a special morphologic and behavioral spectrum. And this opinion is also supported by the discovery of individual immunohistochemical markers of some subtypes such as FoxN1 and CD205 in TSCC, and translocation of the MAML2 gene in mucoepidermoid carcinoma (6, 7).

Reports about prognosis and treatment of thymic carcinomas have increased these years including some large population-based studies (8, 9). Some of them proposed that histologic category, complete resection, great vessel invasion, or adjuvant treatment have effects on prognosis in thymic carcinomas. However, few reports were published for only subtype analysis owing to the low incidence (10). Most of the current reports included various subtypes that covered up the inherent clinicopathologic characteristics and prognostic factors of each histologic subtype.

Using the Surveillance, Epidemiology, and End Results (SEER) database, our study identified a cohort of 276 patients with TSCC. We undertook this retrospective analysis to extract clinicopathological features, treatments, and survival outcomes of TSCC and define disease characteristics and prognostic factors for this rare disease.

## Materials and Methods

### Patient Population

**Figure 1:**
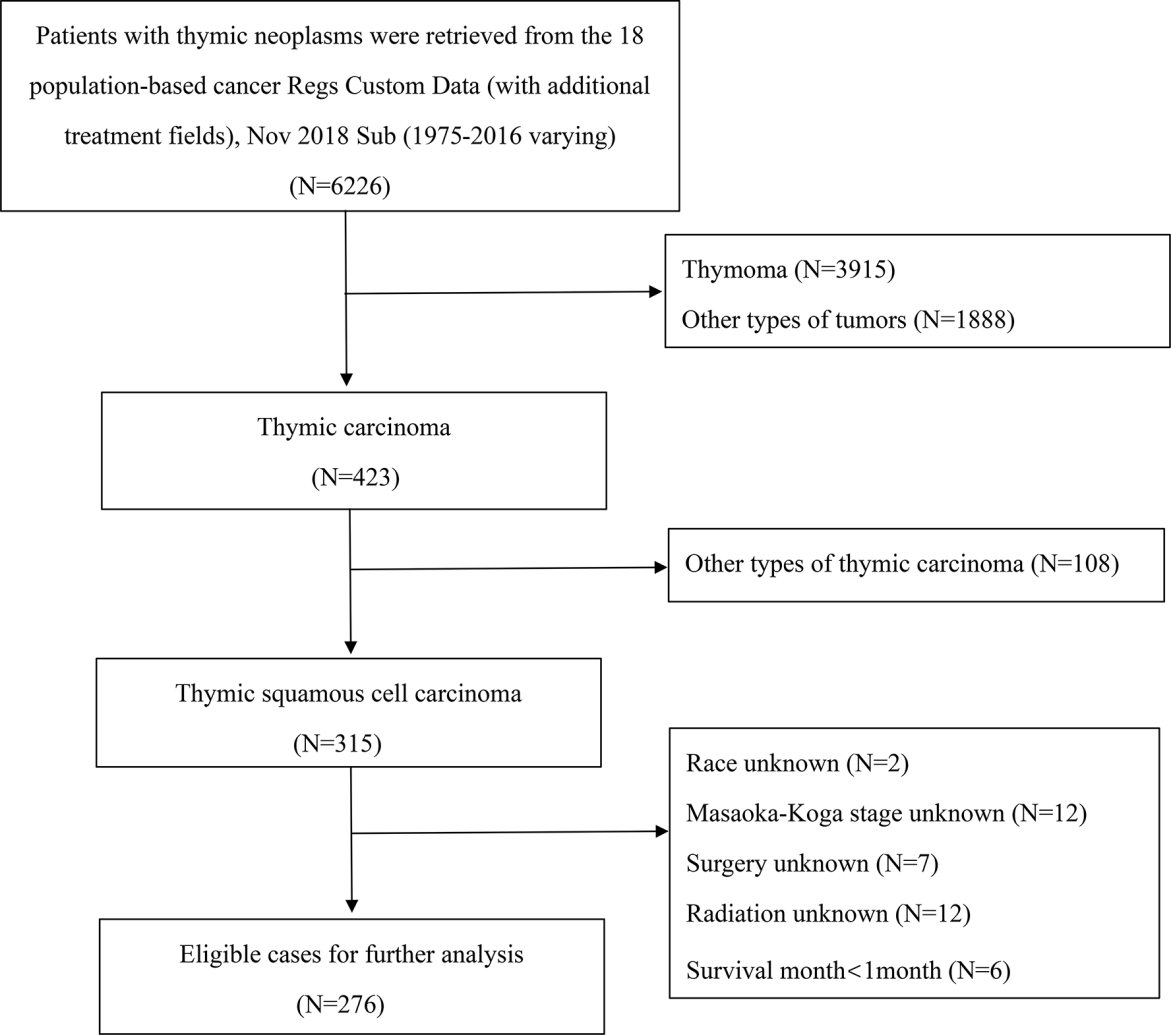
Selection flowchart for patients with TSCC.

The SEER database is sponsored by the National Cancer Institute of American and encompasses the demographics, survival data, and treatments of ~28% of the American population. Our study used the SEER-18 Dataset including 18 cancer registries across the United States for this analysis. Patients with thymic tumors diagnosed from January 1, 1975, to December 31, 2016, were included in this study. The first patient with TSCC was not recorded in the database until 1990. Data were extracted using the SEER*Stat software (version 8.3.6) of the National Cancer Institute. Histologically diagnosed cases were identified by the specific codes of the International Classification of Diseases for Oncology, 3^rd^ edition (ICD-O-3) for the following: (I) primary sites: C37.9 (thymus); (II) histological codes, 8070-8074/3 (TSCC). Data regarding demographics, Masaoka-Koga stage grade, tumor size, nodal metastasis, surgery types (no surgery; incomplete resection including local excision, debulking, and partial removal; complete resection including total resection and radical surgery), radiotherapy, and chemotherapy were obtained. Patients without information on race, Masaoka-Koga stage, surgery, and radiotherapy were excluded. Cases with survival time <1 month were also excluded for the elimination of immediate perioperative mortality (**Figure 1**). The Masaoka-Koga stage was classified into I/IIA (grossly and microscopically completely encapsulated/microscopic transcapsular invasion), IIB (macroscopic invasion into thymic or surrounding fatty tissue, or grossly adherent to but not breaking through mediastinal pleura or pericardium), III (macroscopic invasion of the neighboring organs such as pericardium, great vessel, or lung), and IV (Iva, pleural or pericardial dissemination; IVb, lymphatic or hematogenous metastasis) in accordance with the extension of the primary site in “localized or organ-confined”, “adjacent connective tissue”, “adjacent organs or structures”, and “further contiguous extension” of the SEER database. Positive lymph nodes metastasis was considered as Masaoka-Koga stage IV (3).

### Statistical Analysis

Student’s *t*-test was used to compare continuous variables and categorical variables were analyzed utilizing the Chi-square test. Overall survival (OS) was the period from diagnosis to death or last follow-up. Kaplan-Meier plots were constructed to estimate OS. Differences in histologic subtypes, stage, and surgery types were evaluated with the log-rank test. Related variables with *p*-value < 0.15 in univariate analysis were included in the multivariate analysis using the Cox proportional hazard model. One-to-one matching propensity score matching (PSM) analysis was used to adjust the interference of confounding factors on chemotherapy and radiotherapy. Gender, age at diagnosis, years of diagnosis, ethnicity, grade, Mssaoka-Koga stage, tumor size, nodal metastasis, surgery types, radiotherapy, and chemotherapy were covariates used for matching. All statistical analyses were performed using SPSS version 23.0 (IBM Inc. Chicago, IL, USA). A two-tailed *p*-value less than 0.05 was considered as statistically significant.

### Ethics

This is a population study that involves no identifiable information for individuals throughout the analyses. Institutional Review Board at West China Hospital approved this study and gave ethics exemption because the study was deemed not to constitute human subject research.

## Results

### Patients Characteristics

The SEER database included 3,915 thymomas, 315 TSCC, 5 basaloid carcinomas, 17 mucoepidermoid carcinomas, 26 lymphoepithelioma-like carcinomas, 4 clear cell carcinomas, 8 sarcomatoid carcinomas, 24 adenocarcinomas, 20 undifferentiated carcinomas, and 4 adenosquamous carcinomas. TSCC formed 7.2% of thymic epithelial neoplasms and 74.5% of thymic carcinomas.

A total of 276 TSCC cases was selected and analyzed (**Table 1**). The number of diagnosed cases increased between the years 2010 and 2016 (153) compared to 2001–2010 (100) and 1990–2000 (23). The median patient age was 65 (range, 24–85) years old. Male was predominant (59.8%) in this disease, and 186 patients were Caucasian. Of the 276 cases, 243 had a record of lymph nodes status, of which 87 showed positive nodal metastasis. Tumor size was recorded in 217 cases—median tumor size was 6.3 cm—of which 74 measured 0–5cm, 127 measured 5–10 cm, and 16 cases measured >10 cm. Masaoka-Koga stage yielded 55 cases with stage I/IIA, 20 with stage IIB, 64 with stage III, and 137 cases with stage IV. 56.5% of the patients received radiotherapy and chemotherapy was applied in 59.1%. The complete and incomplete resection was performed in 32.6 and 26.8% of the patients, respectively. Chemotherapy alone (17.4%) was the most common treatment regimen, followed by complete resection + chemoradiotherapy (15.6%) and chemoradiotherapy (15.2%).

**Table 1:** Clinical characteristics of patients (n = 276) with TSCC in the SEER database.

Features	Number (%)
**Gender**	
Male	165 (59.8)
Female	111 (40.2)
**Age at diagnosis (years)**	
Median (range)	65 (24-89)
Mean	64 ± 13
>65	135 (48.9)
≤65	141 (51.1)
**Years of diagnosis**	
1990-2000	23 (8.3)
2001-2010	100 (36.2)
2010-2016	153 (55.4)
**Ethnicity**	
Caucasian	186 (67.4)
African American	30 (10.9)
Asian	60 (21.7)
**Grade**	
Well	15 (8.4)
Moderate	28 (15.7)
Poor	129 (72.5)
Undifferentiated	6 (3.4)
Unknown	98
**Tumor size (cm)**	
Median	6.3(0.1-99.0)
Mean	6.4 ± 2.8
0-5	74 (34.1)
5-10	127 (58.5)
>10	16 (7.4)
Unknown	59
**Nodal metastasis**	
Yes	87 (35.8)
No	156 (64.2)
Unknown	33
Masaoka-Koga stage	
I/IIA	55 (19.9)
IIB	20 (7.2)
III	64 (23.2)
IV	137 (49.6)
**Surgery types**	
Complete resection (radical surgery/total resection)	90 (32.6)
Incomplete resection (local excision/partial removal/debulking)	74 (26.8)
No surgery	112 (40.6)
**Radiotherapy**	
Yes	156 (56.5)
No	120 (43.5)
**Chemotherapy**	
Yes	163 (59.1)
No/Unknown	113 (40.9)
**Treatment regimens**	
NO	15 (5.4)
Chemotherapy alone	48 (17.4)
Radiotherapy alone	7 (2.6)
Chemoradiotherapy	42 (15.2)
Complete resection alone	18 (6.5)
Incomplete resection alone	26 (9.4)
Complete resection + chemotherapy	7 (2.5)
Complete resection + radiotherapy	22 (8.0)
Complete resection + chemoradiotherapy	43 (15.6)
Incomplete resection + chemotherapy	6 (2.2)
Incomplete resection + radiotherapy	25 (9.1)
Incomplete resection + chemoradiotherapy	17 (6.2)

Stage III/IV showed a trend towards larger tumor size (p = 0.052), older age (p = 0.002), and chemotherapy (p < 0.001). Patients with stage I/IIA were more likely to undergo surgery (p < 0.001), and higher proportion of complete resection was performed in patients with stage I//IIA (p < 0.001). The other comparative analysis revealed that patients with smaller tumor size (p = 0.018), negative nodal metastasis (p < 0.001), Masaoka-Koga stage I/II (p < 0.001) and radiotherapy (p < 0.001) were more likely to undergo cancer-directed surgery while patients without surgery were more likely to receive chemotherapy (p < 0.001) (**Table 2**).

**Table 2:** Characteristics of TSCC patients divided by Masaoka-Koga stage and surgery.

Features	I/IIN (%)	III/IVN (%)	p value	NO Surgery N (%)	Surgery N (%)	P value
**Total**	75 (100)	201 (100)		112 (100)	164 (100)	
**Gender**			0.154			0.215
Male	50 (66.7)	115 (57.2)		62 (55.4)	103 (62.8)	
Female	25 (33.3)	86 (42.8)		50 (44.6)	61 (37.2)	
**Age at diagnosis (years)**			0.150			0.354
>65	42 (56.0)	93 (46.3)		51 (45.5)	84 (51.2)	
≤65	33 (44.0)	108 (53.7)		61 (54.5)	80 (48.8)	
Mean	67 ± 11	63 ± 14	**0.002**	63 ± 14	65 ± 12	0.363
**Years of diagnosis**			0.120			0.721
1990-2000	8 (10.7)	15 (7.5)		8 (7.1)	15 (9.1)	
2001-2010	20 (26.7)	80 (39.8)		39 (34.8)	61 (37.2)	
2010-2016	47 (62.7)	106 (52.7)		65 (58)	88 (53.7)	
**Ethnicity**			0.473			0.696
Caucasian	47 (62.5)	139 (69.2)		74 (66.1)	112 (68.3)	
African American	8 (10.7)	22 (10.9)		11 (9.8)	19 (11.6)	
Asian	20 (26.7)	40 (19.9)		27 (24.1)	33 (20.1)	
**Grade**			0.261			0.478
Well	5 (10.9)	10 (7.6)		5 (7.5)	10 (9.0)	
Moderate	10 (21.7)	18 (13.6)		9 (13.4)	19 (17.1)	
Poor	31 (67.4)	98 (74.2)		49 (73.1)	80 (72.1)	
Undifferentiated	0	6 (4.5)		4 (6.0)	2 (1.8)	
Unknown	29	69		45	53	
**Tumor size (cm)**			0.052			**0.001**
0-5	31 (45.6)	43 (28.9)		12 (16.9)	62 (42.5)	
5-10	33 (48.5)	94 (63.1)		53 (74.6)	74 (50.7)	
>10	4 (5.9)	12 (8.1)		6 (8.5)	10 (6.8)	
Unknown	7	52		41	18	
Mean	5.7 ± 3.2	7.3 ± 8.0	0.111	7.0 ± 2.4	6.1 ± 3.0	**0.018**
**Nodal metastasis**			**<0.001**			**<0.001**
Yes	0	87 (50)		42 (46.7)	112 (74.7)	
No	69 (100)	87 (50)		49 (53.3)	38 (25.3)	
Unknown	6	27		21	12	
**Masaoka-Koga stage**						**<0.001**
I/IIA				9 (8.0)	46 (28.0)	
IIB				1 (0.9)	19 (11.6)	
III				25 (22.3)	39 (23.8)	
IV				77 (68.8)	60 (36.6)	
**Surgery types**			**<0.001**			
Complete resection	34 (35.3)	40 (19.9)				
Incomplete resection	31 (41.3)	59 (29.4)				
No surgery	10 (13.3)	102 (50.7)				
**Radiotherapy**			0.704			**<0.001**
Yes	41 (54.7)	115 (57.2)		49 (43.8)	107 (65.2)	
No	34 (45.3)	86 (42.8)		63 (56.3)	57 (34.8)	
**Chemotherapy**						**<0.001**
Yes	19 (25.3)	144 (71.6)	**<0.001**	90 (80.4)	73 (44.5)	
No/Unknown	56 (74.7)	57 (28.4)		22 (19.6)	91 (55.5)	

Bold p-values meant the statistical results were significant.

### Survival Analysis

**Figure 2:**
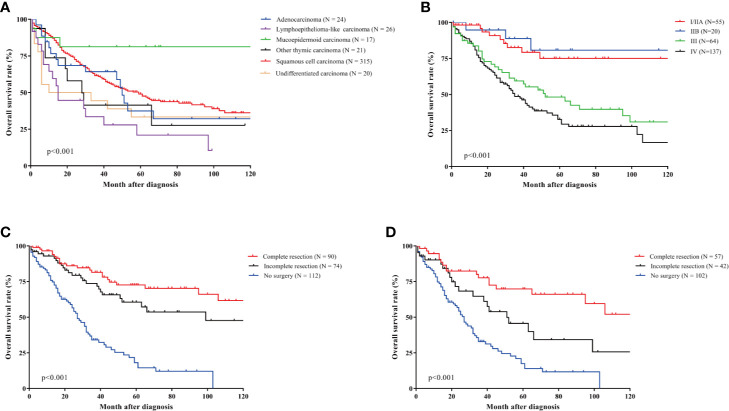
Survival curves of thymic carcinomas according to subtypes, Masaoka-Koga stage, and surgery types. **(A)** Survival curves of thymic carcinomas according to subtypes. Significant survival differences were observed between TSCC and other thymic carcinomas patients. The 5-year OS rates of TSCC were 49.0%. Mucoepidermoid carcinoma performed the best survival outcomes with 81.3% 5-year OS rate, and lymphoepithelioma-like carcinoma had lowest 5-year OS rate (32.1%). **(B)** Survival curves of TSCC according to Masaoka-Koga stage. Masaoka-Koga stage was optimal for TSCC prognosis prediction (p < 0.001). The 5-year OS rates of patients with stage I/IIA, IIB, III and IV were 75.1%, 80.7%, 48.2%, 32.7%, respectively. **(C)** Survival curves of TSCC according to surgery types. Complete and incomplete resection improved survival time obviously (p < 0.001). The 5-year OS rate of complete resection, incomplete resection, and no surgery was 72.7%, 60.6%, and 18.1%, respectively. **(D)** Survival curves of TSCC patients with Masaoka-Koga stage III/IV according to surgery types. Complete resection had the best survival time (69.8% 5-year OS rate), and the 5-year OS rate of incomplete resection and no surgery was 45.6% and 17.5%, respectively (p < 0.001).

Kaplan-Meier curves showed significant differences between thymic carcinomas subtypes (p < 0.001) (**Figure 2A**). The median OS duration of TSCC patients was 59 months and the 5- OS rate was 49.0%. Mucoepidermoid carcinoma performed the best survival outcomes with an 81.3% 5-year OS rate, and lymphoepithelioma-like carcinoma had the lowest 5-year OS rate (32.1%). Undifferentiated carcinomas also showed a worse survival (33.3% 5-year OS rate) than TSCC. Masaoka-Koga stage was optimal for TSCC prognosis prediction (p < 0.001). The 5-year OS rates of patients with stage I/IIA, IIB, III, and IV were 75.1, 80.7, 48.2, and 32.7%, respectively (**Figure 2B**). Complete and incomplete resection improved survival time obviously (p < 0.001). The 5-year OS rate of complete resection, incomplete resection, and no surgery was 72.7, 60.6, and 18.1%, respectively (**Figure 2C**). However, there was no significant survival difference between complete and incomplete resection (p = 0.148). For patients with Masaoka-Koga stage III/IV, Complete resection was the preferred treatment with a 69.8% 5-year OS rate, and the 5-year OS rate of incomplete resection and no surgery was 45.6 and 17.5%, respectively (p < 0.001). The 5-year OS rates of other variables were listed in **Table 3**.

**Table 3:** Univariate and multivariate analysis for patients with TSCC.

Variables	5-year survival rates (%)	Univariate		Multivariate	
		HR (95% CI)	p	HR (95% CI)	p
**Gender**			**0.136**		0.285
Female	40.5	Reference			
Male	50.6	0.76(0.529-1.09)	**0.136**	0.810(0.550-1.192)	0.285
**Age at diagnosis (years)**			0.419		
≤65	50.7	Reference			
>65	45.6	1.16(0.809-1.663)	0.419		
**Years of diagnosis**					
1990-2000	39.5	Reference	0.736		
2001-2010	48.2	0.839(0.475-1.48)	0.544		
2010-2016	52.7	0.788(0.435-1.43)	0.434		
**Ethnicity**			0.789		
Caucasian	50.0	Reference			
African American	40.3	1.219(0.691-2.149)	0.494		
Asian	45.5	1.01(0.64-1.592)	0.966		
**Grade**			0.187		
Well	39.5	Reference			
Moderate	37.1	0.939(0.402-2.196)	0.885		
Poor	43.8	0.72(0.342-1.515)	0.386		
Undifferentiated	20.8	2.024(0.606-6.764)	0.252		
**Tumor size (cm)**			**0.019**		0.178
0-5	52.5	Reference			
5-10	51.2	1.169(0.723-1.887)	0.524	0.769(0.457-1.294)	0.323
>10	20.2	2.153(1.086-4.269)	**0.028**	1.608(0.783-3.303)	0.196
**Nodal metastasis**			**<0.001**		0.613
No	59.3	Reference			
Yes	30.4	2.205(1.489-3.266)	**<0.001**	1.123(0.657-1.92)	0.672
**Masaoka-Koga stage**			**<0.001**		**0.003**
I/IIA	75.1	Reference			
IIB	80.7	0.7(0.189-2.587)	0.593	0.892(0.237-3.356)	0.865
III	45.4	3.319(1.588-6.939)	**0.001**	3.247(1.503-7.015)	**0.003**
IV	29.4	4.575(2.286-9.155)	**<0.001**	3.884(1.674-9.014)	**0.002**
**Surgery types**			**<0.001**		**<0.001**
Incomplete resection	60.6	Reference			
Complete resection	18.1	3.240(2.034-5.161)	**<0.001**	2.844(1.578-5.126)	**<0.001**
No surgery	72.7	0.644(0.365-1.135)	**0.128**	0.554(0.305-1.006)	0.052
**Chemotherapy**			**0.030**		**0.014**
No/unknown	57.7	Reference			
Yes	41.2	1.519(1.041-2.214)	**0.030**	0.555(0.347-0.886)	**0.014**
**Radiotherapy**			0.255		
No	43.7	Reference			
Yes	51.5	0.811(0.565-1.163)	**0.255**		

Bold p-values meant the statistical results were significant.

### Prognosis Analysis

Outcomes of the Cox proportional hazard model analysis for identifying significant potential prognostic factors are depicted in **Table 3**. Univariate analysis showed tumor size >10 cm, positive nodal metastasis, Masaoka-Koga stage III, stage IV, and chemotherapy were risk factors. Conversely, complete resection was a protective factor. Age, gender, ethnicity, diagnosis years histological grade, and radiotherapy had no significant effects on prognosis. The risk factor—chemotherapy in univariate analysis changed to the protective factor in multivariate analysis (HR = 0.555, 95% CI 0.347–0.886; p = 0.014). Complete resection was also the independent protective factor (HR = 2.844, 95% CI 1.578–5.126; p **<** 0.001). Conversely, Masaoka-Koga stage III (HR = 3.247, 95% CI 1.503–7.015; p = 0.003) and stage IV (HR = 3.884, 95% CI 1.674–9.014; p = 0.002) were independent risk factors.

### Survival Analysis After Propensity Score Matching

**Figure 3:**
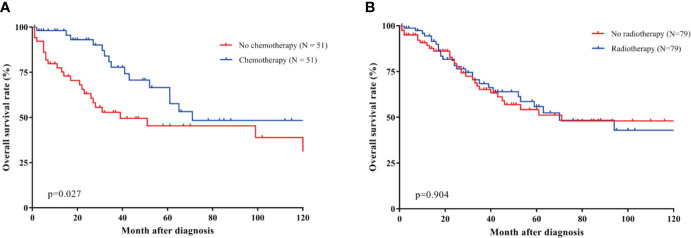
PSM analysis for survival benefits of chemotherapy and radiotherapy in patients with TSCC. **(A)** Significant survival differences were observed between patients with or without chemotherapy (p = 0.027). The 3-year OS rate was 77.7% and 52.8%, respectively. **(B)** Radiotherapy didn’t bring survival benefits (p = 0.904).

Converse results appeared in the prognosis analysis of chemotherapy. To verify the effects of chemotherapy and radiotherapy, we performed PSM based on variables described in materials and methods. Regular data showed that chemotherapy showed a trend towards high-grade (p = 0.008), large tumor size (p < 0.001), positive nodal metastasis (p < 0.001), advanced Massaka-Koga stage (p < 0.001), and no surgery (p < 0.001) (**Table 4**). And compared to patients without radiotherapy, patients with radiotherapy were more likely to undergo surgery (p < 0.001), be > 65 years old (p < 0.006), and chemotherapy-free (p = 0.015) (**Supplementary Table 1**). No significant differences of baseline features were observed in chemotherapy and radiotherapy groups that including 51 and 79 matched patients, respectively. Chemotherapy showed a trend for prolonging survival time (p = 0.027), and the 3-year OS rate was 77.7% and 52.8%, respectively (**Figure 3A**). However, radiotherapy showed no survival benefits (p = 0.904) (**Figure 3B**).

**Table 4:** Baseline characteristics of TSCC patients divided by chemotherapy in the regular and matched groups.

Features	No chemotherapy N (%)	Chemotherapy N (%)	p value	No chemotherapy N (%)	Chemotherapy N (%)	p value
**Total**	113 (100)	163 (100)		51 (100)	51 (100)	
**Gender**			0.698			0.537
Male	66 (58.4)	99 (60.7)		31 (47.7)	34 (52.3)	
Female	47 (41.6)	64 (39.3)		20 (39.2)	17 (33.3)	
**Age at diagnosis (years)**			**0.009**			0.692
>65	66 (58.4)	69 (42.3)		23 (45.1)	25 (49.0)	
≤65	47 (41.6)	94 (57.7)		28 (54.9)	26 (51.0)	
**Years of diagnosis**			0.228			0.064
1990-2000	13 (11.5)	10 (6.1)		7 (13.7)	2 (3.9)	
2001-2010	37 (32.7)	63 (38.7)		13 (25.5)	23 (45.1)	
2010-2016	63 (55.8)	90 (55.2)		31 (60.8)	26 (51.0)	
**Ethnicity**			0.160			0.612
Caucasian	71 (62.8)	115 (70.6)		29 (56.9)	29 (56.9)	
African American	17 (15.0)	13 (8.0)		8 (15.7)	5 (9.8)	
Asian	25 (22.1)	35 (21.5)		14 (27.5)	17 (33.3)	
**Grade**			**0.008**			0.432
Well	7 (6.2)	8 (4.9)		3 (5.9)	1 (2.0)	
Moderate	17 (15.0)	11 (6.7)		9 (17.6)	6 (11.8)	
Poor	42 (37.2)	87 (53.4)		20 (39.2)	23 (45.1)	
Undifferentiated	0 (0.0)	6 (3.7)		0 (0)	2 (3.9)	
Unknown	47 (41.6)	51 (31.3)		19 (37.3)	19 (37.3)	
**Tumor size (cm)**			**<0.001**			0.641
0-5	45 (39.8)	29 (17.8)		15 (29.4)	14 (27.5)	
5-10	43 (38.1)	84 (51.5)		20 (39.2)	24 (47.1)	
>10	8 (7.1)	8 (4.9)		5 (9.8)	2 (3.9)	
Unknown	17 (15.0)	42 (25.8)		11 (21.6)	11 (21.6)	
**Nodal metastasis**			**<0.001**			0.853
No	81 (71.7)	75 (46.0)		28 (54.9)	30 (58.8)	
Yes	18 (15.9)	69 (42.3)		14 (27.5)	14 (27.5)	
Unknown	14 (12.4)	19 (11.7)		9 (17.6)	7 (13.7)	
**Masaoka-Koga stage**			**<0.001**			0.968
I/IIA	43 (38.1)	12 (7.4)		9 (17.6)	8 (15.7)	
IIB	13 (11.5)	7 (4.3)		5 (9.8)	4 (7.8)	
III	28 (24.8)	36 (22.1)		15 (29.4)	15 (29.4)	
IV	29 (25.7)	108 (66.3)		22 (43.1)	24 (47.1)	
**Surgery types**			**<0.001**			0.412
No surgery	22 (19.5)	90 (55.2)		18 (35.3)	21 (41.2)	
Incomplete resection	51 (45.2)	23 (14.1)		17 (33.3)	11 (21.6)	
Complete resection	40 (35.4)	50 (30.7)		16 (45.7)	19 (54.3)	
**Radiotherapy**			0.339			0.321
No	53 (46.9)	67 (41.1)		21 (41.2)	26 (51.0)	
Yes	60 (53.1)	96 (58.9)		30 (58.8)	25 (49.0)	

Bold p-values meant the statistical results were significant.

## Discussion

Most published reports focused on the overall assessment of thymic carcinomas due to the paucity of patients. Only Zhao et al. showed a large population-based study with 105 TSCC cases in 2013 (10). Shimosato et al. and Weissferdt et al. also summarized the clinicopathologic features of TSCC only in small cases (11, 12). We assume each subtype of thymic carcinomas has its special clinical features and progression behaviors. Study results of the published thymic carcinoma reports were usually inconstant and even contradictory due to the different proportions of subtypes. There is an urgent need of the study unique to each subtype with a large population for all-round and deep recognition of thymic carcinomas. In the present study, we analyzed data from the largest cohort of 276 cases with TSCC retrieved from the database. Our findings may present an objective perspective on the characteristics and prognosis of TSCC.

The proportion of TSCC 74.5% in thymic carcinomas identified in the SEER database was in concordance with some previous reports (61.8–73.4%) (4, 10, 13). Some clinical features of TSCC in our study were also in agreement with previously published findings including a higher number of males, median tumor size of 7.0 cm, and more patients who presented with advanced stage III/IV (1, 13–15). Asymptomatic appearance is the leading cause of more advanced stages. In contrast to age at diagnosis peaking between the fifth to sixth decade (16, 17) described in the literature, TSCC patients in the SEER database showed a peak above the sixth decade.

The 5-year OS rate identified in the current study (49.0%) was in accordance with literature reports—33.3 to 65% (14, 16, 18–22) for thymic carcinomas—decreasing to 40.3% at 8-years and 36.2% at 10-years. Our data firstly found that mucoepidermoid carcinoma had the best survival and lymphoepithelioma-like carcinoma was the most malignant subtypes. Undifferentiated carcinoma also showed a worse prognosis than TSCC. Interestingly, Zhao et al., who showed less aggressive behavior associated with TSCC than with other thymic carcinomas due to similarities in the 5-year (59.5%) and 8-year (54.5%) OS rates (10), 5% decrease rate over 3-year, which is in agreement with the 8.3% decrease from 5-year to 8-year in our study. Existence after 5-year may mean long-term survival for TSCC patients.

It is imperative that patients who undergo treatment not only achieve local and total control of tumors but also maintain physiological functions and quality of life. Treatment paradigms comprising surgery have considerable effect on patient survival. In the absence of complex extension and metastasis at the time of diagnosis, total resection is recommended, which was considered as the determinant prognostic factor for thymic carcinoma patients in many studies (8, 9, 13, 20, 23). Our study also demonstrated that complete resection had the best survival outcome.

Radiotherapy and chemotherapy were recommended for TSCC patients with incomplete resection and for patients with advanced stage who are considered unfit for surgery. Improved survival after postoperative radiotherapy was showed by Lim et al. in thymic carcinomas (24). However, we showed that radiotherapy had no effect on extending survival time whether in the regular or matched groups. and this opinion was also supported by some previous reports (4, 13, 16). Since radiotherapy is recommended for incomplete resection or unresectable patients, it is plausible that the controversial findings in survival noted between studies may be due to the omission of comorbidities that correlate to increased mortality, which may have resulted in patients with poor prognosis after receiving radiotherapy, thereby masking the benefits of radiotherapy.

Some reports also showed controversial findings of chemotherapy effects in thymic carcinoma (4, 10, 14, 25). Chemosensitivity was different between subtypes of thymic carcinomas. Zhao et al. found a significant survival benefit of chemotherapy in TSCC patients (10). Our study proposed this high chemosensitivity again due to the survival improvement verified by both multivariate Cox proportional hazard model and PSM analysis. TSCC may be more sensitive to chemotherapy than other thymic carcinomas subtypes.

Though the Masaoka-Koga system is an effective tool to predict the prognosis of thymoma, its utility for survival analysis of thymic carcinoma remains inconclusive. Whereas prognosis using the Masaoka-Koga system was validated in some studies (4, 15, 26, 27), others reported the lack of it (10, 13, 19, 28), which may be due to the small sample size of these studies as well as the heterogeneity of thymic carcinomas subtypes. Though we retrieved a large sample size of 276 patients from the SEER database, the inability to differentiate between Masaoka-Koga stage I and IIA or classifying Masaoka-Koga stage IV as stage IVa and IVb, are limitations in the SEER database. Nevertheless, we found through our multivariate analysis that Masaoka-Koga stage III and stage IV were independent risk factors and were applicable to TSCC. Moreover, Stage IIB showed a better 5-year OS rate than stage I/IIA in our study, which may be caused by a smaller number of cases (20) in stage IIB compared with Stage I/IIA (55). Similar survival between stage IIA and stage IIB also eliminated the difference. The official system—TNM-based system was proposed by the International Thymic Malignancies Interest Group (ITMIG) and the International Association for the Study of Lung Cancer (IASLC) in 2014 (29). However, listing the SEER data according to this system was impossible because of the data absence in T- and N-stage. The TNM stage would be applied in our ongoing studies.

Despite histological grade being a crucial prognostic factor in several tumors, as demonstrated in high-grade and low-grade thymic carcinomas (13, 21), our univariate analysis showed a lack of differences in survival between TSCC patients with different histological grades. This may be attributed to the loss of information on histological differentiation in 35.5% of TSCC cases in the SEER database.

The use of the SEER database for a comprehensive and objective interpretation of clinical features and prognosis for TSCC patients has certain limitations. First, Analyses of the influence of histological differentiation, tumor size, and nodal metastasis on prognosis were limited due to the lack of data. Some reports suggested that tumor size and nodal metastasis had an effect on prognosis (1, 27). Since tumor size was unknown in 59 patients and nodal metastasis record was missing for 33 patients in the SEER database, multivariate analysis to determine prognosis was limited in our study.

Second, different treatment regimens including various agents have been evolving according to different guidelines in the long-term (26-year) interval. But it was worth mentioning that only 23 cases were diagnosed in the period from 1990-2000. Bias induced by a small number of cases may be negligible. Besides, Many studies aiming at thymic carcinomas had a long-term research period, which seems to be the inevitable limitation due to the paucity of the disease (14, 19).

Third, lack of data on various chemotherapy regimens, surgery types (sternotomy, VATS, or RAST), and clinical symptoms in the SEER database was a limitation to determine their influences on the prognosis of TSCC patients in our study.

Lastly, the lack of data on disease-free survival and response to treatment in the SEER database limited our ability to comprehensively analyze these items.

## Conclusions

TSCC was frequently diagnosed in older patients with advanced Masaoka-Koga stage and more favorable survival than other subtypes of thymic carcinomas. Complete resection is the preferred treatment. Masaoka-Koga stage and chemotherapy had a strong association with prognosis.

## Data Availability Statement

The original contributions presented in the study are included in the article/**Supplementary Material**; further inquiries can be directed to the corresponding author.

## Author Contributions

LL and XY conceived and designed the study. Administrative support was done by KZ. CL provided the study materials and patients. YY collected and assembled the data. CG and YP analyzed and interpreted the data All authors wrote and gave the final approval of the manuscript. All authors contributed to the article and approved the submitted version.

## Funding

The study was supported by the Natural Science Foundation of China (81602025) and 1.3.5 Project for Disciplines of Excellence and West China Hospital, Sichuan University (ZYGD18021).

## Conflict of Interest

The authors declare that the research was conducted in the absence of any commercial or financial relationships that could be construed as a potential conflict of interest.
