# Supplementary material for: Thymic Squamous Cell Carcinoma: A Population-Based Surveillance, Epidemiology, and End Result Analysis
Source: Front Oncol. 2020 Dec 22;10:592023. doi: 10.3389/fonc.2020.592023 (PMC7783386; doi:10.3389/fonc.2020.592023)
Supplement: Supplementary file 1 [file Table_1.docx]

**Supplementary table 1** Characteristics of TSCC patients divided by radiotherapy in the regular and matched groups.

| **Features** | **No radiation**  **N (%)** |  | **Radiation**  **N (%)** | **p value** |  | **No radiation**  **N (%)** |  | **Radiation**  **N (%)** | **p value** |
| --- | --- | --- | --- | --- | --- | --- | --- | --- | --- |
| **Total** | 120 (100) |  | 156 (100) |  |  | 79 (100) |  | 79 (100) |  |
| **Gender** |  |  |  | 0.498 |  |  |  |  | 1.000 |
| Male | 69 (57.5) |  | 96 (61.5) |  |  | 37 (46.8) |  | 37 (46.8) |  |
| Female | 51 (42.5) |  | 60 (38.5) |  |  | 42 (53.2) |  | 42 (53.2) |  |
| **Age at diagnosis (years)** |  |  |  | **0.006** |  |  |  |  | 0.746 |
| >65 | 50 (41.7) |  | 91 (58.3) |  |  | 33 (41.8) |  | 31 (39.2) |  |
| ≤65 | 70 (58.3) |  | 65 (41.7) |  |  | 46 (58.2) |  | 48 (60.8) |  |
| **Years of diagnosis** |  |  |  | 0.064 |  |  |  |  | 0.926 |
| 1990-2000 | 9 (7.5) |  | 14 (9.0) |  |  | 5 (6.3) |  | 6 (7.6) |  |
| 2001-2010 | 35 (29.2) |  | 65 (41.7) |  |  | 29 (36.7) |  | 30 (38.0) |  |
| 2010-2016 | 76 (63.3) |  | 77 (49.4) |  |  | 45 (57.0) |  | 43 (54.4) |  |
| **Ethnicity** |  |  |  | 0.821 |  |  |  |  | 0.757 |
| Caucasian | 83 (69.2) |  | 112 (66) |  |  | 55 (69.6) |  | 52 (65.8) |  |
| African American | 13 (10.8) |  | 17 (10.9) |  |  | 8 (10.1) |  | 11 (13.9) |  |
| Asian | 24 (20.0) |  | 36 (23.1) |  |  | 16 (20.3) |  | 16 (20.3) |  |
| **Grade** |  |  |  | 0.888 |  |  |  |  | 0.323 |
| Well | 7 (5.8) |  | 8 (5.1) |  |  | 5 (6.3) |  | 1 (1.3) |  |
| Moderate | 10 (8.3) |  | 18 (11.5) |  |  | 5 (6.3) |  | 9 (11.4) |  |
| Poor | 48 (40.0) |  | 81 (51.9) |  |  | 36 (45.6) |  | 37 (46.8) |  |
| Undifferentiated | 2 (1.7) |  | 4 (2.6) |  |  | 1 (1.3) |  | 3 (3.8) |  |
| Unknown | 53 (44.2) |  | 45 (29.4) |  |  | 32 (40.5) |  | 29 (36.7) |  |
| **Tumor size (cm)** |  |  |  | 0.409 |  |  |  |  | 0.413 |
| 0-5 | 32 (26.7) |  | 42 (27.5) |  |  | 22 (27.8) |  | 22 (27.8) |  |
| 5-10 | 50 (41.7) |  | 77 (50.3) |  |  | 39 (49.4) |  | 32 (40.5) |  |
| >10 | 4 (3.3) |  | 12 (7.8) |  |  | 2 (2.5) |  | 6 (7.6) |  |
| Unknown | 34 (28.3) |  | 25 (16.3) |  |  | 16 (45.7) |  | 19 (54.3) |  |
| **Nodal metastasis** |  |  |  | 0.687 |  |  |  |  | 0.187 |
| No | 69 (57.5) |  | 87 (56.9) |  |  | 53 (67.1) |  | 45 (57.0) |  |
| Yes | 36 (30.0) |  | 51 (33.3) |  |  | 15 (19.0) |  | 25 (31.6) |  |
| Unknown | 15 (12.5) |  | 18 (11.8) |  |  | 11 (13.9) |  | 9 (11.4) |  |
| **Masaoka-Koga stage** |  |  |  | 0.089 |  |  |  |  | 0.289 |
| I/IIA | 29 (24.2) |  | 26 (16.7) |  |  | 24 (30.4) |  | 16 (20.3) |  |
| IIB | 5 (4.2) |  | 15 (9.6) |  |  | 4 (5.1) |  | 9 (11.4) |  |
| III | 23 (19.2) |  | 41 (26.3) |  |  | 18 (22.8) |  | 21 (26.6) |  |
| IV | 63 (52.5) |  | 74 (47.4) |  |  | 33 (41.8) |  | 33 (41.8) |  |
| **Surgery types** |  |  |  | **<0.001** |  |  |  |  | 0.798 |
| No surgery | 63 (52.5) |  | 49 (31.4) |  |  | 29 (36.7) |  | 25 (31.6) |  |
| Incomplete incision | 32 (26.8) |  | 42 (26.9) |  |  | 28 (35.4) |  | 30 (38.0) |  |
| Complete incision | 25 (20.8) |  | 65 (41.7) |  |  | 22 (27.8) |  | 24 (30.4) |  |
| **Chemotherapy** |  |  |  | **0.015** |  |  |  |  | 0.524 |
| No | 59 (49.2) |  | 54 (34.6) |  |  | 40 (50.6) |  | 36 (45.6) |  |
| Yes | 61 (50.8) |  | 102 (65.4) |  |  | 39 (49.4) |  | 43 (54.4) |  |
